# Effect of myocardial Scar detected by Cardiac Magnetic Resonance on perfusion time and short term outcomes after Coronary Artery Bypass Graft Surgery

**DOI:** 10.1186/1532-429X-18-S1-P85

**Published:** 2016-01-27

**Authors:** Kalyan Kancherla, Krishna Kancharla, Gaby Weissman, Abdalla A Elagha, Swetha Samineni, Peter C Hill, Steven Boyce, Anthon Fuisz

**Affiliations:** 1 Cardiology, MedStar Health Research Institute, Washington, DC USA; 2 Cardiology, Mayo Clinic, Rochester, MN USA; 3 Cardiology, MedStar Heart and Vascular Institute, MedStar Washington Hospital Center, Washington, DC USA; 4 Cardiology, Georgetown University, Washington, DC USA; 5 Translational Medicine Branch, National Heart, Lung, and Blood Institute, Bethesda, MD USA; 6 Cardiology, Cairo University Hospitals, Cairo, Egypt; 7 Mayo Clinic Health Systems, Austin, MN USA; 8 Cardiac Surgery, MedStar Heart and Vascular Institute, MedStar Washington Hospital Center, Washington, DC USA; 9 Cardiology, Westchester Medical Center, Valhalla, NY USA

## Background

Myocardial viability assessment by late gadolinium enhancement (LGE) on Cardiac Magnetic resonance (CMR) is an important prognostic tool in patients undergoing CABG. We aim to evaluate the association of scar with surgical parameters and 30 day outcomes post CABG as defined by Society of Thoracic Surgeons (STS) database.

## Methods

Patients who underwent LGE CMR between January 2003 and February 2010 <1 month prior to CABG were included. A standard 16 segment model was used for Left ventricular (LV) scar quantification. Patients were categorized into Scar group and No-scar groups. Perfusion time (PT), Cross clamp time (CCT), 30-Day mortality, Ventricular arrhythmia, duration of ventilation, prolonged ICU (ICU-LOS) and hospital stay (H-LOS) were obtained. Chi square test, t- test, Wilcoxon rank sum tests and multivariate regression analysis was used for data analysis.

## Results

196 patients met the inclusion criteria. 185 CMR studies were available. The median time from CMR study to CABG surgery was 2 (1, 4) days. The mean age of the study population was 63.2 years (± 11.5). Seventy-two percent were male. History of prior MI was present in 64% of patients and prior CABG in 5.4% patients. Median LV ejection fraction was 38% (28, 52). Cardiopulmonary bypass was used in 118 patients (72%).

There were 133 patients (72%) in the scar group and 52 patients (38%) in the No-scar group. Compared with No-scar group, Scar group has higher proportion of men 78% Vs 56% (p = 0.002), History of prior MI 74% Vs 39% (P = 0.0001) and lower LVEF 39% Vs 46 (P = 0.038).

Perfusion time (70 ± 19 min Vs 60 ± 15 min, P = 0.01) and Cross clamp time (48 ± 14 min Vs 40 ± 13 min, P = 0.004) were significantly longer in Scar group. In multivariate model, number of vessels bypassed (p < 0.0001), presence of scar (p = 0.0075), Age (p = 0.047) and lower LVEF (p = 0.049) were independently associated with longer perfusion time. Number of vessels bypassed (p < 0.0001), presence of scar (p = 0.0009) were independently associated with longer cross clamp time. There was no significant difference in 30-Day mortality, arrhythmia, duration of ventilation, prolonged ICU and hospital stay.

## Conclusions

In patients undergoing surgical revascularization, presence of myocardial scar is significantly associated with longer perfusion and cross clamp time independent of traditional predictors. However, there is no significant difference in short term postoperative outcomes based on scar.

**Table 1:** Short Term Outcomes

Parameters	All patients Mean / Median(IQ)/%	No-scar Mean / Median(IQ)/%	Scar group Mean/Median(IQ)/%	P value
Number of patients	185	52	133	
Perfusion Time (min)	67.3 (± 18.4)	60.4 (± 14.9)	69.8 (± 19.0)	0.0129
Cross Clamp Time (min)	45.98 (± 13.7)	40.1 (± 12.5)	48.3 (± 13.5)	0.0041
Short term Mortality (%)	4.9 (n = 9)	5.8	4.5	0.71
Arrhythmias (%)	13.0	15.4	12.0	0.54
Total Ventilator Hours	9.1 (5.9, 19.3)	9 (5.8, 17.0)	9.7 (5.9, 19.8)	0.84
Prolonged ICU-LOS (%)	29.67	30.8	29.2	0.74
Prolonged H-LOS (%)	16.2	17.6	15.6	0.74

